# Respiratory failure, underlying acute illnesses, and hospital outcomes: the S. Giovanni-Addolorata–SIGOT GRACE Study

**DOI:** 10.1007/s11739-025-04074-3

**Published:** 2025-08-24

**Authors:** Filippo Luca Fimognari, Angelo Scuteri, Elena Del Giudice, Francesco Baffa Bellucci, Umberto Giuseppe Galasso, Andrea Cavalli, Mariagiovanna Cozza, Elvira Clausi, Lorenzo Palleschi

**Affiliations:** 1 Unit of Geriatrics, Dipartimento Medico Polispecialistico, Azienda Ospedaliera “Annunziata-Mariano Santo-S. Barbara” di Cosenza, Via Felice Migliori, 87100 Cosenza, Italy; 2 Dipartimento Scienze Mediche e Sanità Pubblica, Università di Cagliari, Cagliari, Italy; 3 Unit of Internal Medicine, Azienda Ospedaliero-Universitaria di Cagliari, Cagliari, Italy; 4 Unit of Geriatrics, Dipartimento Internistico con Area Onco-Ematologica, Azienda Ospedaliera S. Giovanni-Addolorata di Roma, Rome, Italy; 5 Intermediate Care/Budrio Long-Term Care Unit, Dipartimento della Integrazione, Azienda Unità Sanitaria Locale (AUSL) di Bologna, Bologna, Italy

**Keywords:** Respiratory failure, Geriatric hospital wards, Hospital mortality, Length of hospital stay, Non-intensive medical patients

## Abstract

Although respiratory failure (RF) is frequent among older medical patients admitted to non-intensive hospital units, inherent data are scarce. We determined whether RF predicted adverse hospital outcomes independently of its causative illnesses. In a retrospective observational study from the Geriatric Risk Assessment and Care Evaluation database, we included 1093 patients consecutively admitted to a geriatric hospital unit (2022–2024). Study outcomes included hospital death, length of hospital stay (LOS) and post-discharge institutionalization (discharge to nursing homes or other long-term facilities). RF was diagnosed according to admission peripheral oxygen saturation ≤ 91%, or oxygen therapy/non-invasive ventilation during hospitalization, or specific diagnostic discharge codes. The predictive role of RF was investigated controlling for RF causative illnesses, frailty measured by the Multidimensional Prognostic Index (MPI), and C-reactive protein. The RF prevalence was 43%. Compared to controls, RF patients had higher hospital mortality (25.4% vs. 6.0%) and longer LOS, but comparable institutionalization rate. The fully adjusted odds ratio (OR) of RF for hospital mortality was 3.98 (95% Confidence Interval [CI] 2.53–6.28) and further increased after exclusion of 106 acute-on-chronic RF patients (4.71, CI 2.96–7.49). MPI, C-reactive protein, and sepsis emerged as additional significant predictors of mortality. RF also predicted longer LOS (F 6.78, p 0.009) in a linear regression model, along with age, MPI, pneumonia, pulmonary embolism, stroke, and sepsis. RF was highly prevalent and predicted hospital mortality and longer LOS *per se*, independently of its causative acute illnesses, frailty, and systemic inflammation. Older patients should be actively screened for RF during hospitalization.

**Supplementary Information:**

The online version contains supplementary material available at 10.1007/s11739-025-04074-3.

## Introduction

Respiratory failure (RF) is a medical condition characterized by the presence of critical hypoxia in the arterial blood. RF is the final outcome of various acute and acute-on-chronic disorders mainly regarding the cardio-respiratory system, such as heart failure, pneumonia, exacerbation of chronic obstructive pulmonary disease, but also sepsis and other systemic diseases [1].

In a nationwide administrative Italian study, RF was the first discharge diagnosis from hospital wards among patients aged ≥ 75 years admitted from the Emergency Department (ED) [2]. RF is therefore supposed to be a highly prevalent acute syndrome among medical older patients hospitalized outside the Intensive Care Units (ICUs). This issue, however, was investigated only by a few studies conducted in small samples of adult subjects, which calculated a prevalence of critical hypoxia of about 10–20% in medical non-intensive hospital units [3–5]. Recently, we reported a RF prevalence of 20% in older patients in medical units enrolled in a nationwide study [6]. In another recent study of patients admitted to a geriatric acute care unit only through the ED, the prevalence rose to 48% [7]. In these studies, RF was associated with frailty and overall severity of acute illness [6] and emerged as an independent predictor of short-term or in-hospital death after accounting for older age, comorbidity, education, recent hospitalization, frailty, delirium, and severity of illness [6, 7]. RF, however, remains a syndrome in which hypoxia is the result of underlying and causative illnesses. In a seminal study of Ray et al., the hospital prognosis of RF older patients significantly improved if the illnesses that caused RF were correctly diagnosed and treated since arrival in the ED [1]. Therefore, RF may not have an independent prognostic role *per se*, but its negative prognostic impact could merely reflect the effect of its triggering illnesses. This issue was never previously investigated.

We analyzed the Geriatric Risk Assessment and Care Evaluation (GRACE) database, jointly developed by S. Giovanni-Addolorata Hospital and SIGOT (Società Italiana di Geriatria Ospedale e Territorio, Italian Society of Hospital and Community Geriatrics). Our aims were to calculate the prevalence of RF in a sample of older patients hospitalized in a geriatric acute care unit and to determine whether the RF syndrome, even mild RF that is treated outside the ICUs, predicted adverse hospital outcomes irrespective of its underlying and associated diseases and of other recognized predictors of risk, such as clinical frailty and systemic inflammation.

## Methods

### The GRACE database

The GRACE is a retrospective observational cohort study of all patients consecutively admitted to the Acute Care Geriatric Unit of the Azienda Ospedaliera S. Giovanni-Addolorata, Rome, Italy. Data collection started in November 2022 and is currently ongoing. The methodology of GRACE study is described in detail elsewhere [8]. For the present study, we analyzed the first enrolled 1093 patients consecutively hospitalized in the Unit from November 2022 to April 2024. Clinical data from admission to discharge were anonymously extracted from the electronic medical records and the Hospital Discharge Record (HDR). Extracted data included 6 categories: (I) socio-demographic variables; (II) department of origin; (III) laboratory measures and vital parameters at admission; (IV) Comprehensive Geriatric Assessment at admission; (VI) diagnoses and procedures coded in the HDR after discharge; and (VII) hospital outcomes.

The department of origin included the ED or other hospital wards. Collected laboratory measures at hospital admission included hemoglobin, C-reactive protein (CRP), and serum creatinine. Vital parameters at admission included systolic blood pressure, heart rate, temperature, and peripheral oxygen saturation measured by pulse oximetry (SpO_2_). Oxygen therapy and a treatment of non-invasive ventilation during hospital stay were also reported in the database.

The Comprehensive Geriatric Assessment was carried out within the first 2 days of hospital stay obtaining the Multidimensional Prognostic Index (MPI), a validated tool for predicting mortality in hospitalized older people and resulting from the score of 8 scales exploring 8 different domains [9], as follows: the Activities of Daily Living scale for measuring the ability to perform the basic activities of living [10]; the Instrumental Activities of Daily Living to assess the ability in instrumental tasks [11]; the 10-item Short Portable Mental Status Questionnaire for investigating cognitive status [12]; the Cumulative Illness Rating Scale for assessing comorbidity [13]; the Short Form of the Mini Nutritional Assessment, designed for a rapid screening of malnutrition [14]; the Exton-Smith Scale for measuring the risk of developing pressure sores [15]; the number of drugs taken by the patient within the first 2 days of hospitalization; and cohabitation status (i.e., living with family, residence in a long-term facility, living alone). The results of these scales were included in a software calculating the final MPI score, ranging from 0 to 1. MPI categorized patients as having low (MPI value ≤ 0.33), moderate (between 0.34 and 0.66), and severe risk (> 0.66) of mortality at 6 and 12 months of follow-up [9]. Dates of hospital admission and discharge, and illnesses coded according to the International Classification of Diseases, 9th Revision, Clinical Modification [ICD-9-CM] were extracted from the HDRs. All HRDs were coded after careful review of medical records by a trained staff physician and then controlled by one of the authors (L.P.). Random controls regarding the matching between codes and diagnoses documented in the medical records were performed at least once a year by medical personnel of the hospital direction.

As this study was based on a secondary analysis of anonymously extracted data, approval of the hospital institutional review board was obtained to transfer clinical data from the electronic applications to a single encrypted database.

### Diagnosis of RF and other illnesses

A monitoring protocol of the study hospital unit requests to measure SpO_2_ at admission and then at least once per day (during the morning round), as well as in occasion of each urgent intervention due to any clinical deterioration. Since diagnostic criteria for diagnosing RF are not absolute [16], we used a broad definition that included in the RF group patients who had at least one of the following conditions: SpO_2_ ≤ 91% at admission measured on room air or during oxygen therapy/non-invasive ventilation; oxygen therapy or non-invasive ventilation during hospital stay; and one or more of the following codes reported in the HRD: 51881, 51882, 5184, 5185, 51884, and 51883.

The ICD-9 codes 51883 and 51884 (acute-on-chronic RF) were attributed to patients who reported long-term oxygen therapy before admission and presented an acute respiratory deterioration since admission or during hospitalization  [7]

The SpO_2_ ≤ 91% cut-off was chosen for the following reasons: this [6, 7] and an even more conservative diagnostic cut-off (SpO_2_ ≤ 92% or partial pressure of oxygen ≤ 70 mmHg on room air) [1] were clinically meaningful, as they were used in previous studies that demonstrated worse hospital outcomes in populations of RF older patients observed in non-intensive settings; when SpO_2_ was measured on room air, this cut-off allowed to capture also patients with mild hypoxemia; conversely, when it was measured in patients treated with oxygen therapy or non-invasive ventilation, it identified patients with moderate-to-severe RF.

The ICD-9-CM codes used to categorize the other illnesses included in the analysis are listed in the Supplementary Table.

### Study outcomes

Length of hospital stay (LOS) was calculated as the difference between the discharge and the admission dates. In-hospital death was defined as death during the stay in the study unit, and being discharged alive was the reference category. The third study outcome was “institutionalization” after hospital discharge, i.e., discharge to long-term facilities, including any long-term care and rehabilitation facility, nursing homes, and hospices, with discharge to home (including discharge against medical advice) as the reference category.

### Analytic strategy and statistical analysis

A set of clinical variables was compared across the two groups with and without RF (univariate analysis). Then, multivariate models were constructed, including clinical variables that were significantly different in RF compared to non-RF patients, in order to determine whether RF was independently associated with the study outcomes that were significantly different between the two groups in the univariate analysis. Initial models included age, sex, MPI, and CRP as covariates. Additional models further included, as covariates, comorbidities with different prevalence across the two groups (pneumonia, pulmonary embolism, pleural effusion, acute heart failure, chronic obstructive pulmonary disease, stroke, lung neoplasms, and sepsis). These models were built both in the total sample (*N* = 1093) and in the subsample (*N* = 987) including only acute RF patients, i.e., obtained excluding the 106 patients with acute-on-chronic RF (codes 51884 or 51883).

Categorical variables are presented as frequencies, while continuous variables as means with standard deviations. Differences of clinical and laboratory variables between patients with and without RF were compared by the *t*-test or the chi-square test, as appropriate. The association between RF and LOS was studied via multivariable regression analysis models. Multivariate logistic regression was used to assess the association of RF with both in-hospital mortality and post-discharge institutionalization. All analyses were performed using SAS on Demand for Academics—SAS Studio version 9.04 (SAS Institute Inc., Cary, NC, USA). All statistical tests were two sided. *P* values < 0.05 were considered significant.

## Results

Table 1 illustrates the characteristics of patients with and without RF and univariate comparisons between the two groups. Patients diagnosed with RF were older and more frequently females. Both LOS (18.8 ± 15.0 versus 14.3 ± 11.2 days) and in-hospital mortality (25.4% versus 6%) were significantly greater in RF patients as compared to those without RF. Sepsis, pneumonia, pulmonary embolism, chronic obstructive pulmonary disease, pleural effusion, heart failure, and lung neoplasms were significantly more prevalent in RF patients, while stroke was less prevalent.

**Table 1:** General and clinical variables in patients with and without respiratory failure

	No repiratory failure (*n* = 617)	Respiratory failure (*n* = 476)	*p* value
Age (years)	84.8 ± 6.9	85.9 ± 7.1	0.019
Women (%)	53.5	63.0	0.031
MPI score < 0.333 (%) 0.333–0.666 (%) > 0.666 (%)	0.558 ± 0.209 18.3 44.6 37.1	0.632 ± 0.202 10.5 37.6 51.9	< 0.0001
CIRS severity	2.0 ± 0.4	2.2 ± 0.5	< 0.0001
CIRS comorbidity	4.1 ± 2.3	4.8 ± 2.5	< 0.0001
Length of stay (days)	14.3 ± 11.2	18.8 ± 15.0	< 0.0001
In-hospital mortality (%)	6.0	25.4	< 0.0001
Post-discharge institutionalization (%)	27.8	30.7	0.34
Body temperature (°C)	36.2 ± 0.4	36.2 ± 0.4	0.23
Systolic blood pressure (mmHg)	137.1 ± 21.1	132.9 ± 22.8	0.0012
Heart rate (beats × minute)	78.6 ± 13.5	81.8 ± 14.5	< 0.0001
SpO_2_ (%)	96.2 ± 1.7	94.9 ± 2.7	< 0.0001
Hemoglobin (g/dl)	11.8 ± 1.9	11.6 ± 2.2	0.11
C-reactive protein (mg/dL)	5.5 ± 5.0	8.3 ± 7.6	< 0.0001
Creatinine (mg/dl)	1.2 ± 1.0	1.3 ± 1.0	0.55
Sepsis (%)	4.8	12.4	< 0.0001
Pneumonia (%)	5.1	28.8	< 0.0001
Pulmonary embolism (%)	1.8	4.1	0.0082
COPD (%)	1.6	12.8	< 0.0001
Pleural effusion (%)	5.4	18.9	< 0.0001
Acute heart failure (%)	13.1	29.3	< 0.0001
Acute coronary syndrome (%)	0.6	0.7	0.84
Stroke (%)	13.7	9.7	0.028
Renal failure (%)	19.2	19.3	0.9
Lung neoplasms (%)	2.1	5.4	0.0012

*MPI* Multidimensional Prognostic Index, *CIRS* Cumulative Illness Rating Scale, *SpO*_*2*_ peripheral oxygen saturation; *COPD* Chronic Obstructive Pulmonary Disease

At admission in the ward, 42 patients had SpO_2_ ≤ 91%; in 35 of these patients, SpO_2_ was measured during oxygen therapy or non-invasive ventilation, while in 7 patients it was measured on room air.

In the subsample of 987 patients obtained removing the 106 patients with acute-on-chronic RF (codes 51884 or 51883), the remaining prevalence of acute RF was 37.5% (370 patients). The in-hospital mortality of these 370 patients with acute RF was 28.9% (107 patients), as opposed to 6% (37 patients) of the 617 patients without RF and to a 25.4% mortality (121 patients) of the total RF group (476 patients) including both acute and acute-on-chronic RF patients.

As illustrated in Table 2, RF was an independent determinant of LOS, together with older age and lower MPI (Table 2, model 1). When controlling for prevalent conditions underlying or associated with RF, the role of RF as an independent determinant of LOS remained virtually unchanged in the total sample (Table 2, model 2) and mostly in the subsample without acute-on-chronic RF patients, i.e., including only those with acute RF (model 3).

**Table 2:** Determinants of length of hospital stay at linear regression analysis models

	Model 1		Model 2		Model 3*	
	*F* value	*p* value	*F* value	*p* value	*F* value	*p* value
Age	9.86	0.0017	11.82	0.0006	11.42	0.0008
Female sex	4.07	0.044	2.97	0.085	5.14	0.024
MPI	19.36	< 0.0001	11.89	0.0006	12.13	0.0005
Respiratory failure	24.75	< 0.0001	6.78	0.0094	7.42	0.0066
C-reactive protein	0.38	0.54	0.30	0.58	0.65	0.42
Pneumonia			13.73	0.0002	10.41	0.0013
Pulmonary embolism			4.40	0.036	2.40	0.12
Acute heart failure			0.53	0.47	1.20	0.27
Stroke			14.86	0.0001	14.25	0.0002
COPD			0.43	0.51	0.54	0.46
Pleural effusion			1.81	0.18	1.93	0.17
Lung neoplasms			1.00	0.32	2.66	0.10
Sepsis			44.39	< 0.0001	40.79	< 0.0001

*MPI* Multidimensional Prognostic Index, *COPD* Chronic Obstructive Pulmonary Disease

^*^In the subsample obtained excluding patients with codes 51884 or 51883, i.e., those with acute-on-chronic RF (who reported long-term oxygen therapy prior to hospital admission)

**Fig. 1:**
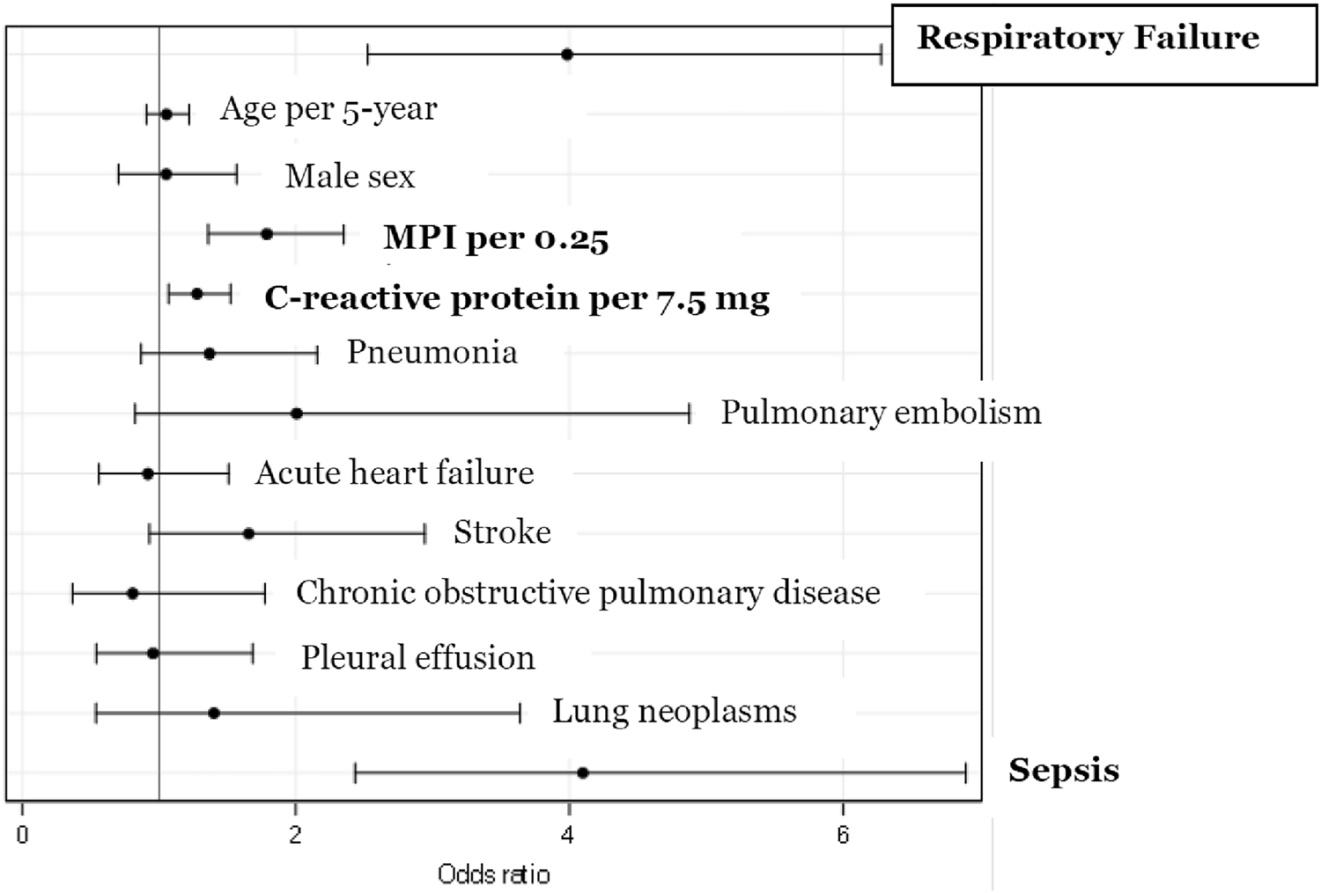
Logistic regression analysis of clinical variables associated with in-hospital mortality. *MPI* Multidimensional Prognostic Index

When investigating the determinants of in-hospital mortality, unadjusted logistic regression showed that RF was associated with a fivefold higher odds of in-hospital mortality (Odds Ratio [OR] 5.34, 95% Confidence Interval [CI] 3.61–7.90, *p* < 0.0001). Three sets of multivariable logistic regression models, including the same covariates as models predicting LOS, were constructed (Table 3). In model 1—including age, sex, MPI, and CRP as covariates—RF was significantly associated with in-hospital death (OR 4.36, 95% CI 2.88–6.61). In model 2—including the same covariates as model 1 plus prevalent conditions underlying and/or associated with the RF syndrome—RF was again strongly associated with in-hospital death (OR 3.98, 95% CI 2.53–6.28), as illustrated in Fig. 1. This risk of death associated with RF increased in model 3, constructed in the subsample including only patients with acute RF (OR 4.71, 95% CI 2.96–7.49).

**Table 3:** Logistic regression analysis models predicting in-hospital mortality

	Model 1			Model 2			Model 3*		
	OR	95% CI	*p* value	OR	95% CI	*p* value	OR	95% CI	*p* value
Respiratory failure	4.36	2.88–6.61	< 0.0001	3.98	2.53–6.28	< 0.0001	4.71	2.96–7.49	< 0.0001
Age (5-year increase)	1.03	0.90–1.18	0.7	1.05	0.91–1.22	0.49	1.06	0.91–1.24	0.47
Male sex	1.13	0.78–1.66	0.52	1.05	0.71–1.57	0.8	0.93	0.60–1.42	0.73
MPI (0.25 increase)	1.95	1.50–2.55	< 0.0001	1.79	1.36–2.35	< 0.0001	1.59	1.20–2.11	0.0011
C-reactive protein (per SD increase, i.e., 7.5 mg/dl)	1.40	1.19–1.65	< 0.0001	1.28	1.07–1.52	0.0068	1.31	1.09–1.58	0.005
Pneumonia				1.37	0.87–2.15	0.17	1.36	0.83–2.23	0.22
Pulmonary embolism				2.01	0.83–4.88	0.12	2.20	0.86–5.60	0.10
Acute heart failure				0.92	0.56–1.51	0.74	0.88	0.51–1.53	0.66
Stroke				1.66	0.93–2.94	0.09	1.75	0.96–3.19	0.07
COPD				0.81	0.37–1.78	0.59	0.42	0.12–1.45	0.17
Pleural effusion				0.96	0.54–1.69	0.88	0.97	0.52–1.80	0.92
Lung neoplasms				1.40	0.54–3.64	0.49	0.99	0.51–5.57	0.19
Sepsis				4.10	2.43–6.90	< 0.0001	3.75	2.15–6.55	< 0.0001

*MPI* Multidimensional Prognostic Index, *SD* standard deviation, *COPD* Chronic Obstructive Pulmonary Disease

^*^In the subsample obtained excluding patients with codes 51884 or 51883, i.e., those with acute-on-chronic RF (who reported long-term oxygen therapy prior to hospital admission)

Post-discharge institutionalization (discharge to nursing homes or any long-term facility of patients discharged alive) was comparable across the two groups with and without RF (Table 1). The unadjusted OR of RF for institutionalization was 1.15 (CI 0.86–1.54).

## Discussion

The main findings of this study are summarized as follows: RF was diagnosed in 43% of hospitalized patients; in-hospital mortality of RF patients was 25.4% as compared with a mortality of 6% in those without RF; the RF syndrome was a strong and independent predictor of in-hospital mortality and longer LOS, also after accounting for MPI, CRP (expression of systemic inflammation) and the illnesses underlying RF; and this predictive risk further raised when patients with acute-on-chronic RF were excluded from analysis.

The prevalence of RF, including both acute RF and acute-on-chronic RF, was 43%, i.e., only slightly lower than the 48% calculated in our recently published administrative study of patients hospitalized from the ED in another geriatric acute care unit from 2014 to 2016 [7]. In the subsample obtained excluding 106 patients with acute-on-chronic RF, the prevalence of acute RF was 37.6%. These observations suggest that RF, also *de novo* RF, is a highly frequent acute syndrome among older patients hospitalized in non-intensive medical wards, affecting almost every second patient. Albeit broadly neglected by the current literature, RF in non-ICU medical settings seems to have a prevalence widely exceeding that of delirium, a frequent and well-recognized geriatric syndrome [6, 17].

We identified a series of cardio-respiratory illnesses that resulted significantly more frequent among RF patients and contributed to cause the RF syndrome (Table 1). Heart failure was the most frequent, followed by pneumonia, pleural effusion, chronic obstructive pulmonary disease, sepsis, lung neoplasms, and pulmonary embolism. Heart failure, chronic obstructive pulmonary disease, pneumonia, and sepsis were the most frequent causes of RF in our recent study conducted in a geriatric hospital unit [7]. Heart failure, pneumonia, chronic obstructive pulmonary disease, and pulmonary embolism, were more frequently encountered in older RF patients studied in a French ED by Ray in 2001–2002 [1]. In an administrative American survey (2001–2009), also including patients admitted to the ICUs, heart failure, pneumonia, sepsis, chronic obstructive pulmonary disease, adult respiratory distress syndrome, and myocardial infarction were—in order of frequency—the disorders underlying RF in persons aged ≥ 85 years [18]. Therefore, in terms of RF etiologies, the results of the present study are consistent with the existing literature. They indicate that RF of older persons hospitalized outside the ICU is the consequence of the complex and heterogeneous combination of various common acute and acute-on-chronic disorders, with a pivotal role played by heart failure [1, 7, 18, 19].

To our knowledge, this is the first study that aimed at understanding whether, in older medical in-patients, the RF syndrome predicted adverse outcome *per se*, i.e., irrespective of its underlying and causative diseases. We provided evidence that RF is a potent and independent risk factor for both in-hospital death and longer LOS, also after adjustment for important and life-threatening medical conditions underlying RF (heart failure, pneumonia, pleural effusion, pneumonia, chronic obstructive pulmonary disease, sepsis, pulmonary embolism, and lung neoplasms). Interestingly, this predictive power was greater when only acute, *de novo* RF—after exclusion of patients with acute-on-chronic RF—was taken into account. RF independently predicted both outcomes also after correction for MPI, a recognized predictor of poor outcomes in hospitalized medical patients, as well as for systemic inflammation as indicated by CRP levels. RF predicted in-hospital mortality together with MPI, CRP, and sepsis (Fig. 1); it predicted increased LOS along with older age, MPI, pneumonia, pulmonary embolism, stroke, and sepsis. We therefore established a reasonable and consistent pattern of risk factors for both longer LOS and in-hospital death in a real-life hospital setting with high RF prevalence.

There are some possible explanations for this evidence that RF predicted adverse outcomes independently of—and overall better than—its underlying diseases. First, unlike persons affected by such illnesses and without RF, those with RF are—by definition—characterized by arterial hypoxemia, the hallmark of RF. Hypoxemia can arise from a particularly complex interplay of the various causative illnesses. This interaction is supposed to achieve a critical threshold of clinical severity, also related to higher prevalence and severity of the underlying illnesses, that determines hypoxemia and poor clinical outcomes [1, 7, 19]. Second, even though hypoxemia was reasonably controlled by oxygen therapy or non-invasive ventilation, it is supposed to produce harms, such as tissue hypoxia with organ dysfunction or failure, platelet and blood clotting activation, systemic inflammation, oxidative stress, secondary polycythemia, pulmonary hypertension, and others [20]. These effects may increase the risk of worse hospital outcomes. Third, hypoxemia (and RF), irrespective of clinical severity, prevalence, and critical combination of the triggering acute diseases, may occur more easily in patients who are frail before hospitalization, as recently demonstrated [6]. In some patients, RF may therefore represent an epiphenomenon of frailty/disability or terminal disease [6].

In this regard, MPI is a widely used measure of frailty and a reliable predictor of short and long-term mortality [9]. As in our population, MPI had an excellent accuracy and calibration in predicting longer LOS and in-hospital mortality in acute care geriatric settings [21]. Notably, in our population RF predicted in-hospital death and longer LOS regardless of MPI. It is possible that RF reflects domains of frailty not entirely captured even by a potent tool as MPI [6]. RF is also an expression of overall severity of the acute illness, a partly different clinical domain than frailty, and thus poorly explored by MPI [6].

Sepsis was associated with adverse outcomes in our hospitalized older medical patients. While the clinical impact of sepsis was somehow expected, our finding that elevated CRP circulating levels independently predicted in-hospital death requests particular comment. Increased CRP levels are expression of systemic and lung inflammation, as well as of inflammation-related instability of atherosclerotic plaques, representing an intriguing link between inflammatory/infectious diseases and atherothrombotic events [22, 23]. High CRP serum values were repeatedly found to herald adverse outcomes in chronic obstructive pulmonary disease  [23], heart failure [24], cardiovascular diseases [22], pulmonary infections [25], and sepsis [26], all conditions underlying RF. An elevated CRP was also associated with in-hospital death in unselected older medical patients [27]. To our knowledge, however, the interplay between RF and systemic inflammation, signaled by increased circulating CRP, was never investigated in the same population. We observed an independent and separate contribution of RF and high CRP to the risk of in-hospital death of our older medical patients, a clinically meaningful result deserving confirmation and further insight.

RF did not predict institutionalization in our discharged alive patients. This can be attributed, at least in part, to the substantial in-hospital mortality of RF patients and is consistent with our recent study [7]. The 25.4% in-hospital mortality of RF patients was comparable to the 23% calculated in the latter study, and far higher than the 6% rate of non-RF patients. When only acute RF was considered, in-hospital mortality was 28.9%. These numbers mean that, in a non-intensive hospital unit, a diagnosis of RF—even mild RF—separated two groups of medical older patients with dramatically different rate of in-hospital death and LOS [6, 7]. Consequently, a strict SpO_2_ monitoring is pivotal to obtain a timely RF diagnosis, in order to identify promptly the pathologic mechanisms generating the syndrome, as well as to prioritize tailored medical interventions for these vulnerable patients [28]. In the study of Ray, an early diagnosis of the diseases underlying RF, obtained since arrival in the ED, was associated with a better prognosis during the subsequent hospital stay [1].

This study had limitations. First, its retrospective nature precludes any firm conclusion. Second, despite the standardized coding method, the diagnosis of illnesses other than RF was based on administrative data, and a selective ICD-9 reporting or mis-reporting cannot be ruled out. Third, data derive from a single hospital and thus generalizability is uncertain. Finally, the GRACE protocol did not include measures of severity of both RF and overall acute illness [8], that are supposed to affect the impact of RF on mortality [6]. In a previous study, however, we found that the RF-related risk of death in non-intensive medical settings remained significant also after correction for a standardized measure of severity of overall acute illness [6].

In conclusion, RF was very common in older persons acutely hospitalized in a non-intensive medical ward. The RF syndrome was a potent risk factor for longer LOS ad in-hospital mortality, also after keeping into account the prognostic value of its underlying and causative diseases. As a clue for identifying and prioritizing more vulnerable patients, RF should be searched for, clinically interpreted, and carefully managed in older patients hospitalized in acute care non-intensive medical units.

## Supplementary Information

Below is the link to the electronic supplementary material.

Supplementary file1 (DOCX 13 KB)

## Data Availability

The GRACE Study database is available upon reasonable request.

## References

[1] Ray P, Birolleau S, Lefort Y et al (2006) Acute respiratory failure in the elderly: etiology, emergency diagnosis and prognosis. Crit Care 10(3):R82. 10.1186/cc4926 16723034 10.1186/cc4926 PMC1550946

[2] Fimognari FL, Lelli D, Landi F, Antonelli Incalzi R (2022) Association of age with emergency department visits and hospital admissions: a nationwide study. Geriatr Gerontol Int 22(11):917–923. 10.1111/ggi.14481 36116913 10.1111/ggi.14481

[3] Foran M, Ahn R, Novik J et al (2010) Prevalence of undiagnosed hypoxemia in adults and children in an under-resourced district hospital in Zambia. Int J Emerg Med 3(4):351–356. 10.1007/s12245-010-0241-5 21373304 10.1007/s12245-010-0241-5 PMC3047821

[4] Singh V, Aziz A, Wakil Q, Sharma BB (2012) Occurrence of hypoxia in the wards of a teaching hospital. Lung India 29(4):329–331. 10.4103/0970-2113.102804 23243345 10.4103/0970-2113.102804 PMC3519017

[5] Bowton DL, Scuderi PE, Haponik EF (1994) The incidence and effect on outcome of hypoxemia in hospitalized medical patients. Am J Med 97(1):38–46. 10.1016/0002-9343(94)90046-9 8030655 10.1016/0002-9343(94)90046-9

[6] Fimognari FL, Tassistro E, Rossi E et al (2024) The interplay among respiratory failure, delirium, frailty and severity of illness in hospitalized older medical patients: a nationwide multicenter observational study. J Frailty Aging 13(4):480–486. 10.14283/jfa.2024.12 39574271 10.14283/jfa.2024.12

[7] Fimognari FL, De Vincentis A, Arone A, Baffa Bellucci F, Ricchio R, Antonelli Incalzi R (2024) Clinical outcomes and phenotypes of respiratory failure in older subjects admitted to an acute care geriatric hospital ward. Intern Emerg Med 19(5):1359–1367. 10.1007/s11739-024-03625-4 38776046 10.1007/s11739-024-03625-4

[8] Palleschi L, Cavalli A, Galasso UG et al (2025) Analysis of predictive factors for hospitalization outcomes in older patients: the Geriatric Risk Assessment and Care Evaluation (GRACE) study protocol by the Azienda Ospedaliera San Giovanni-Addolorata (AOSGA) and the Italian Society of Hospital and Community Geriatrics (SIGOT). Geriatric Care 11:1. 10.4081/gc.2025.13727

[9] Pilotto A, Ferrucci L, Franceschi M et al (2008) Development and validation of a multidimensional prognostic index for one-year mortality from comprehensive geriatric assessment in hospitalized older patients. Rejuvenation Res 11(1):151–161. 10.1089/rej.2007.0569 18173367 10.1089/rej.2007.0569 PMC2668166

[10] Katz S, Downs TD, Cash HR, Grotz RC (1970) Progress in development of the index of ADL. Gerontologist 10(1):20–30. 10.1093/geront/10.1_part_1.20 5420677 10.1093/geront/10.1_part_1.20

[11] Lawton MP, Brody EM (1969) Assessment of older people: self-maintaining and instrumental activities of daily living. Gerontologist 9(3):179–186 5349366

[12] Pfeiffer E (1975) A short portable mental status questionnaire for the assessment of organic brain deficit in elderly patients. J Am Geriatr Soc 23(10):433–441. 10.1111/j.1532-5415.1975.tb00927.x 1159263 10.1111/j.1532-5415.1975.tb00927.x

[13] Linn BS, Linn MW, Gurel L (1968) Cumulative illness rating scale. J Am Geriatr Soc 16(5):622–626. 10.1111/j.1532-5415.1968.tb02103.x 5646906 10.1111/j.1532-5415.1968.tb02103.x

[14] Rubenstein LZ, Harker JO, Salvà A, Guigoz Y, Vellas B (2001) Screening for undernutrition in geriatric practice: developing the short-form mini-nutritional assessment (MNA-SF). J Gerontol A Biol Sci Med Sci 56(6):M366–M372. 10.1093/gerona/56.6.m366 11382797 10.1093/gerona/56.6.m366

[15] Bliss MR, McLaren R, Exton-Smith AN (1966) Mattresses for preventing pressure sores in geriatric patients. Mon Bull Minist Health Public Health Lab Serv 25(Nov):238–268 6013600

[16] Chen L, Rackley CR (2024) Diagnosis and epidemiology of acute respiratory failure. Crit Care Clin 40(2):221–233. 10.1016/j.ccc.2023.12.001 38432693 10.1016/j.ccc.2023.12.001

[17] Morandi A, Di Santo SG, Zambon A et al (2019) Delirium, dementia, and in-hospital mortality: the results from the Italian Delirium Day 2016, a national multicenter study. J Gerontol A Biol Sci Med Sci 74(6):910–916. 10.1093/gerona/gly154 29982365 10.1093/gerona/gly154

[18] Stefan MS, Shieh MS, Pekow PS et al (2013) Epidemiology and outcomes of acute respiratory failure in the United States, 2001 to 2009: a national survey. J Hosp Med Feb; 8(2):76-82. 10.1002/jhm.2004 23335231 10.1002/jhm.2004 PMC3565044

[19] Murru V, Belfiori E, Sestu A, Casanova A, Serra C, Scuteri A (2025) Patterns of comorbidities differentially impact on in-hospital outcomes in heart failure patients. BMC Geriatr May 23;25(1):371. 10.1186/s12877-025-06002-8 40410665 10.1186/s12877-025-06002-8 PMC12100837

[20] Kent BD, Mitchell PD, McNicholas WT (2011) Hypoxemia in patients with COPD: cause, effects, and disease progression. Int J Chron Obstruct Pulmon Dis 6:199–208. 10.2147/COPD.S10611 21660297 10.2147/COPD.S10611 PMC3107696

[21] Volpato S, Bazzano S, Fontana A, Ferrucci L, Pilotto A, MPI-TriVeneto Study Group (2015) Multidimensional prognostic index predicts mortality and length of stay during hospitalization in the older patients: a multicenter prospective study. J Gerontol A Biol Sci Med Sci 70(3):325–331. 10.1093/gerona/glu167 25209253 10.1093/gerona/glu167 PMC4351394

[22] Ridker PM (2003) Clinical application of C-reactive protein for cardiovascular disease detection and prevention. Circulation Jan 28;107(3):363-9. 10.1161/01.cir.0000053730.47739.3c 12551853 10.1161/01.cir.0000053730.47739.3c

[23] Dahl M, Vestbo J, Lange P, Bojesen SE, Tybjaerg-Hansen A, Nordestgaard BG (2007) C-reactive protein as a predictor of prognosis in chronic obstructive pulmonary disease. Am J Respir Crit Care Med 175(3):250–255. 10.1164/rccm.200605-713OC 17053205 10.1164/rccm.200605-713OC

[24] Albar Z, Albakri M, Hajjari J, Karnib M, Janus SE, Al-Kindi SG (2022) Inflammatory markers and risk of heart failure with reduced to preserved ejection fraction. Am J Cardiol 167:68–75. 10.1016/j.amjcard.2021.11.045 34986991 10.1016/j.amjcard.2021.11.045

[25] Qiao L, Yuan H (2025) Prognostic value of C-reactive protein levels in pulmonary infections: a systematic review and meta-analysis. Medicine (Baltimore) 104(12):e41722. 10.1097/MD.0000000000041722 40128046 10.1097/MD.0000000000041722 PMC11936577

[26] Daud M, Khan MB, Qudrat QU, Ullah I, Khan S, Khan MZ, Yousuf I, Ahmad F (2024) Role of C-reactive protein and procalcitonin in early diagnostic accuracy and their prognostic significance in sepsis. Cureus 16(9):e70358. 10.7759/cureus.70358 39469363 10.7759/cureus.70358 PMC11513552

[27] Nouvenne A, Ticinesi A, Lauretani F et al (2016) The prognostic value of high-sensitivity C-reactive protein and prealbumin for short-term mortality in acutely hospitalized multimorbid elderly patients: a prospective cohort study. J Nutr Health Aging 20(4):462–468. 10.1007/s12603-015-0626-526999249 10.1007/s12603-015-0626-5

[28] Lamberti JP (2020) Respiratory monitoring in general care units. Respir Care 65(6):870–881. 10.4187/respcare.07405 32457176 10.4187/respcare.07405

