# Supplementary material for: Respiratory failure, underlying acute illnesses, and hospital outcomes: the S. Giovanni-Addolorata–SIGOT GRACE Study
Source: Intern Emerg Med. 2025 Aug 24;20(7):2131–9. doi: 10.1007/s11739-025-04074-3 (PMC12534294; doi:10.1007/s11739-025-04074-3)
Supplement: Supplementary file 1 — Supplementary file1 (DOCX 13 KB) [file 11739_2025_4074_MOESM1_ESM.docx]

**Supplementary Table. Codes used to diagnose specific illnesses included in the analysis**

| **Diagnosis** | **ICD-9 code** |
| --- | --- |
| Respiratory Failure | 51881, 51882, 51884, 51883, 5184, 5185 |
| Pneumonia | 480*, 481*, 482*,483*,484*,485*,486*, 507*, 487.0 |
| Sepsis | 78552, 7907, 99590, 99591, 99592, 0380* |
| Chronic obstructive pulmonary disease | 490, 491*, 492*, 496 |
| Pleural effusion | 510*, 511*, 512* |
| Pulmonary embolism | 415* |
| Heart failure | 416*, 428*, 429*, 424.0, 424.1, 424.2, 424.3, 425*, 402.01, 402.11, 402.91, 404*, 421* |
| Acute coronary syndrome | 410*, 411*, 413* |
| Cerebrovascular acute diseases (stroke) | 430, 431, 432*, 43301, 43311, 43321, 43331, 43381, 43391, 43401, 43411, 43491, 435*, 436, 4371, 4372, 4378, 4379 |
| Lung neoplasms | 162*, 163*, 164*, 165*, 1714, 2357, 2358, 2359, 1970, 1971, 1972, 1973, 2391 |
| Renal failure | 584*, 585*, 586 |

*Indicates that all codes with that initial number were considered for diagnosis.
